# The efficacy and safety of acupuncture for cerebral vasospasm after subarachnoid hemorrhage: study protocol for a randomized controlled trial

**DOI:** 10.1186/s13063-015-0591-7

**Published:** 2015-02-28

**Authors:** Seung-Yeon Cho, Dong-Hyuk Lee, Hee Sup Shin, Seung Hwan Lee, Jun Seok Koh, Woo-Sang Jung, Sang-Kwan Moon, Jung-Mi Park, Chang-Nam Ko, Ho Kim, Seong-Uk Park

**Affiliations:** Department of Cardiology and Neurology, College of Korean Medicine, Kyung Hee University, 26 Kyungheedae-ro, Dongdaemun-gu, Seoul 130-701 Republic of Korea; Department of Neurosurgery, College of Medicine, Kyung Hee University, 26 Kyungheedae-ro, Dongdaemun-gu, Seoul 130-701 Republic of Korea; Department of Epidemiology and Biostatistics, Graduate School of Public Health & Institute of Health and Environment, Seoul National University, 1 Gwanak-ro, Gwanak-gu, Seoul 151-742 Republic of Korea; Stroke & Neurological Disorders Center, Kyung Hee University Hospital at Gangdong, 892 Dongnam-ro, Gangdong-gu, Seoul 134-727 Republic of Korea

**Keywords:** Subarachnoid hemorrhage, Vasospasm, Delayed ischemic neurological deficit, Acupuncture

## Abstract

**Background:**

Subarachnoid hemorrhage (SAH) is a neurological disease with a high mortality rate. Several serious complications frequently arise after successful surgery for this condition. Cerebral vasospasm, one such complication, occurs in 50 to 70% of SAH patients. These patients suffer neurological symptoms known as delayed ischemic neurological deficit (DIND); however, the effect of treatment of vasospasm is limited. The major pathogenesis of cerebral vasospasm is the reduction of nitric oxide (NO) and activation of vasoconstrictors. Acupuncture is known to increase the production and activity of vascular endothelial cell-derived NO and improve endothelium-dependent vasodilatation. A preliminary retrospective case study to investigate the ability of acupuncture to prevent the occurrence of cerebral vasospasm has been conducted. However, no randomized, controlled clinical trials have been carried out to evaluate the efficacy of acupuncture for cerebral vasospasm.

**Methods/Design:**

This trial will be a single-center, randomized, placebo-controlled, parallel group, patient-assessor-blinded clinical trial. A total of 80 patients with SAH will be randomized into two groups: a study group given acupuncture, electroacupuncture, and intradermal acupuncture, and a control group given mock transcutaneous electrical nerve stimulation and sham intradermal acupuncture. Intervention will start within 96 h after SAH, and a total of 12 sessions will be performed during a 2-week period. The primary outcome measure will be the occurrence of DIND, and the secondary outcomes will be vasospasm as measured by cerebral angiography, transcranial Doppler, clinical symptoms, vasospasm-related infarcts, NO and endothelin-1 plasma levels, mortality, and modified Rankin Scale scores.

**Discussion:**

This trial will examine the efficacy and safety of acupuncture for cerebral vasospasm after SAH. The placebo effect will be excluded and the mechanism of action of the treatments will be evaluated through blood testing.

**Trial registration:**

ClinicalTrials.gov NCT02275949, Registration date: 26 October 2014.

## Background

Subarachnoid hemorrhage (SAH) can occur spontaneously or as a result of trauma [1]. The most common cause of spontaneous SAH is aneurysm rupture (about 80% of cases), while vascular malformations, tumors, venous angioma, or infectious aneurysms may also cause bleeding [2,3]. The incidence rate is about 1 per 10,000 people per year, and SAH accounts for about 5% of stroke patients [3,4]. The rate of SAH is low when compared to cerebral hemorrhage or cerebral infarction; however, this condition is a large burden to both individual patients and society because of high mortality rates and treatment costs in neurological intensive care units [5-7].

Treatment of SAH involves early ligation of the aneurysm or insertion of a coil; however, complications such as cerebral vasospasm, rebleeding, or hydrocephalus frequently occur after successful surgery [2-4,7,8]. Such complications increase the mortality rate of SAH or lead to patient sequelae [4]. In particular, cerebral vasospasm is the most serious complication of SAH [9]. It is reported that 23% of the deaths or disabilities that occur as a result of SAH are due to vasospasm [10]. Cerebral vasospasm begins 3 to 4 days after the onset of SAH and reaches a maximum between 7 and 10 days [7,11,12]. Vasospasm occurs in 50 to 70% of SAH patients, and about 50% of these patients suffer neurological symptoms such as deterioration of consciousness and language disorders [3,11,13]. Such symptoms are known as delayed ischemic neurological deficit (DIND). Cerebral infarction occurs in half of DIND patients and a permanent neurological deficit remains in about 35%. In addition, 30% of patients die of DIND [4,7].

Spontaneous vasoconstriction and vasodilatation are adjusted by the physiological regulation of vascular endothelial cells [14]. If a cerebral aneurysm ruptures and cerebral blood flow enters the subarachnoid space, hemoglobin in the blood increases the production of reactive oxygen species (ROS) [15]. This triggers secretion of the vasoconstrictor substances endothelin-1, prostaglandin H2, and thromboxane A2 from vascular endothelial cells and decreases levels of the vasodilatory substance nitric oxide (NO) [6,9,10,15,16]. It is known that cerebral vasospasm occurs via this mechanism. In particular, NO and hemoglobin combine very easily; thus, the presence of hemoglobin results in the loss of vasodilatory function [16]. To prevent vasospasm on the basis of its pathogenesis, various methods such as calcium channel blockers, magnesium sulfate, statin agents, endothelin antagonists, and nicardipine prolonged release implants have been used [2,3,7]. However, their effects are limited and various studies of new drugs and treatments are now under way [4,9,10].

Acupuncture is an effective and safe treatment method that has been used for the treatment of acute stroke including SAH for centuries [17]. In recent years, a number of studies have been published that show that acupuncture increases the production and activity of vascular endothelial cell-derived NO and can improve the function of damaged vascular endothelial cells [18,19]. Acupuncture point ST36 (Zusanli) is known to lower blood pressure and sympathetic nervous tension [18,19]. In particular, it was reported that electroacupuncture stimulation at ST36 lowers blood pressure by increasing the activity of endothelial NO [19]. NO released from endothelial cells plays a key role in maintaining the tension of the vascular wall [20]. Since reduction of NO is the major pathogenesis of cerebral vasospasm after SAH, it can be assumed that acupuncture at ST36 will stimulate the production of NO and normalize endothelial function in patients with SAH. Another acupoint, PC6 (Neiguan), is known to reduce the ischemic heart damage caused by increased myocardial oxygen demand as a result of sympathetic nervous excitement; it also lowers blood pressure and reduces nausea and vomiting [21]. In a study using a finger photoplethysmogram, acupuncture at PC6 was reported to be effective in reducing arterial stiffness in hypertensive patients [22]. Although this study was performed to investigate the acute reaction to acupuncture, it can be assumed that acupuncture at PC6 normalized endothelial cell function and so reduced arterial stiffness.

Based on previous reports, a clinical study was conducted in hypertensive patients with damaged arterial endothelial cell function, which confirmed that acupuncture at ST36-PC6 improved endothelium-dependent vasodilatation [23]. After this, we carried out a preliminary retrospective case study in patients with SAH. The results indicated the possibility of acupuncture treatment in preventing the occurrence of cerebral vasospasm after SAH [24]. To confirm the effectiveness and clinical application of acupuncture for cerebral vasospasm, rigorous clinical trials such as randomized, double-blind trials are required. The aim of this study will be to assess the efficacy and safety of acupuncture treatments in SAH patients for preventing delayed cerebral vasospasm through a randomized controlled trial.

## Methods

This study will be a single-center, randomized, placebo-controlled, parallel group, patient-assessor-blinded clinical trial. The study will be conducted at Kyung Hee University Hospital in Gangdong, Seoul, Korea.

### Ethics

The trial will be carried out in accordance with the Declaration of Helsinki and the Korean Good Clinical Practice Guidelines and has been approved by the ethical committee of the Kyung Hee University Hospital at Gangdong (KHNMC-OH-IRB 2014-001-002). It has been registered on www.clinicaltrials.gov (NCT02275949) and will be reported in compliance with the CONSORT statement (www.consort-statement.org).

### Participants

#### Subject enrollment, randomization, and blinding

**Figure 1:**
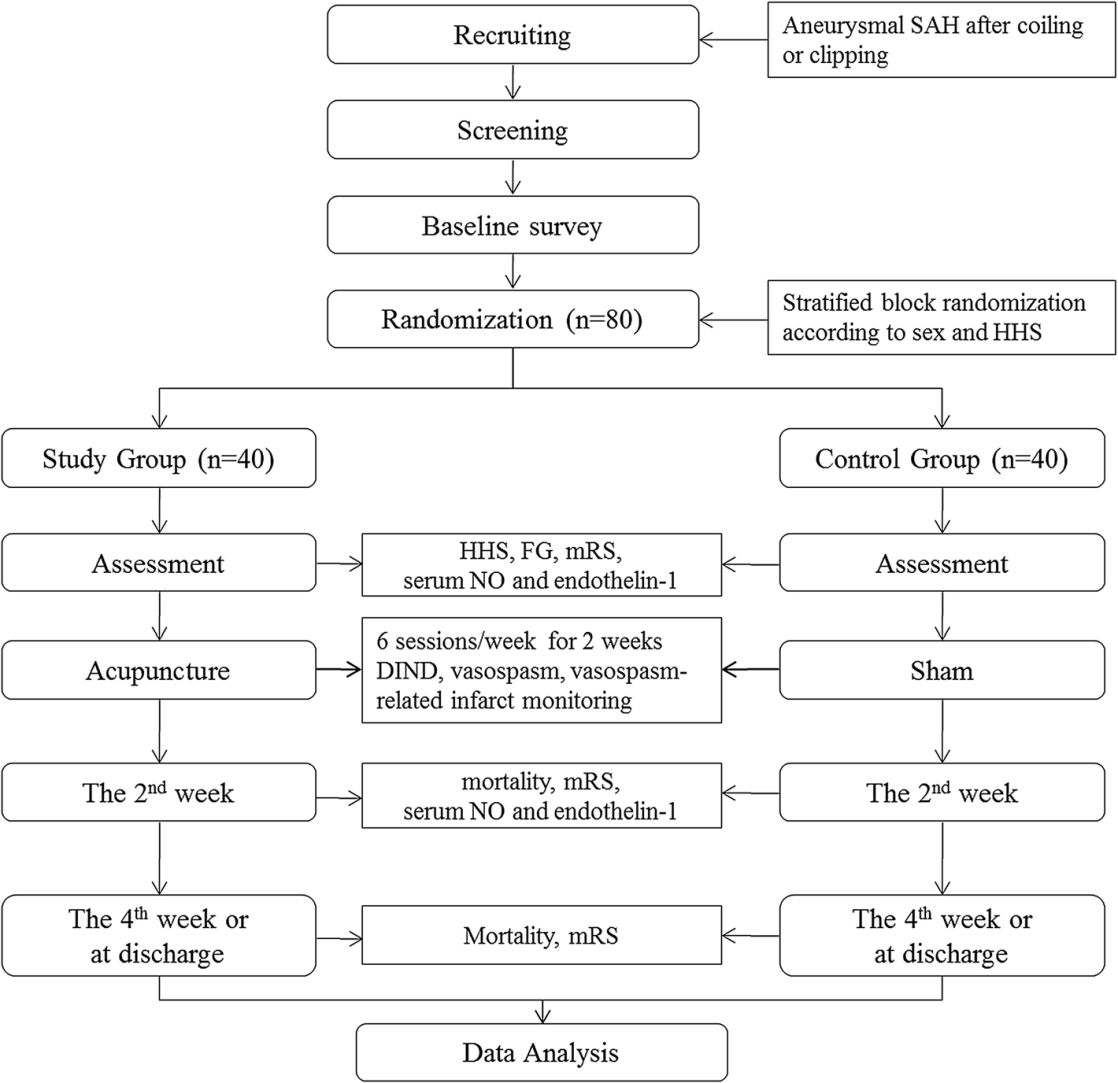
**The flow chart.** HHS, the Hunt and Hess Scale; FG, the Fisher grade; mRS, modified Rankin Scale; NO, nitric oxide; DIND, delayed ischemic neurological deficit.

A total of 80 participants will be recruited for this trial. Patients with SAH admitted to our hospital are potential candidates for the study. Eligible participants will be randomized to the study group or the control group after written informed consent is obtained. Concealed allocation will be achieved by randomization and allocation by an assigned researcher without patient contact. Randomization will be done using a computer-generated allocation list by an assigned researcher not involved in the intervention or assessment. Stratified block randomization will be carried out according to sex and the Hunt and Hess Scale (HHS) [25]. The physician involved in the intervention will receive a random number by phone after the informed consent form has been signed and the HHS has been evaluated. A flow chart of the study is presented in Figure 1.

The participants and the outcome assessors will be blinded to the type of intervention used. The practitioner will not provide any clues about the allocation information to the participants during the study. Blinding will be maintained until the study is completed.

#### Inclusion criteria

Participants conforming to all the following conditions will be included: (1) SAH verified by computed tomography (CT) and cerebral angiography; (2) aneurysm treated by endovascular coiling or surgical clipping; (3) age > 18 years; (4) HHS 1 to 4; (5) acupuncture treatment can start within 96 h after SAH; and (6) participation is voluntary and the informed consent form is signed.

#### Exclusion criteria

Participants with any of the following conditions will be excluded: 1) traumatic or infectious SAH; 2) HHS 5; 3) transcranial Doppler (TCD) cannot be performed; 4) heart, liver, or renal failure; 5) presence of cardiac pacemaker; or 6) have experience of electro-acupuncture.

### Intervention

Intervention will start within 96 h of SAH, after ruptured aneurysms have been secured by endovascular coiling or surgical clipping. Interventions will be applied once a day, 6 days a week for 2 weeks (a total of 12 sessions) in addition to standard treatments such as prophylactic hypertensive, hypervolemic, and hemodilution (triple-H) therapy and nimodipine. Sterile disposable stainless steel acupuncture needles (length, 4 cm; diameter, 0.25 mm) will be used. All interventions will be performed by one Korean Medicine doctor with over 3 years of working experience who completed a college education of 6 years. This doctor will be trained in the study protocol before the start of the trial.

#### Study group

Acupuncture, electroacupuncture, and intradermal acupuncture will be performed at every session. Acupuncture needles (Dong Bang Acupuncture Inc., Chungnam, Korea) will be inserted bilaterally at the acupuncture points Zusanli (ST36), Neiguan (PC6), Gongsun (SP4), and Xiangu (ST43). After insertion to a depth of approximately 1.5 cm, the needles will be manually stimulated until *de qi* (a subjective experience in which patients feel a radiating sensation considered indicative of effective needling) is achieved.

An electric stimulator (ES-160, ITO Co., Japan) will be connected to the handle of each needle in the ST36 and PC6 positions and a current of 5 Hz applied. The current intensity will be increased until light muscle contraction is evident and reaches approximately 70% of bearable intensity. The needles will be left in place for 20 min and then removed. The practitioner will be able to regulate the intensity in response to requests from the subjects.

After the needles are removed, intradermal acupuncture needles with tape (DB130A, 0.25 mm × 1.5 mm, Dong Bang Acupuncture Inc., Chungnam, Korea) will be inserted bilaterally in the ST36, PC6, SP4, and ST43 positions and maintained until the next session.

#### Control group

Mock transcutaneous electrical nerve stimulation and sham intradermal acupuncture will be carried out by the same Korean Medicine doctor. After an electrical insulator (Medi trace 200 foam electrodes, Ludlow Technical Products Canada, Ltd., Ontario, Canada) is attached bilaterally in the ST36 and PC6 positions, the same electric stimulator will be connected to the same points as used in the study group, but without application of current. The subjects in the control group will see the same lights and hear the same sounds coming from the machine as the study group. After 20 min, the electrical insulator will be removed and sham intradermal acupuncture will be performed at the same points. 3 M micropore medical tape (1 cm × 1 cm) will be placed on the skin. After intradermal acupuncture needles are placed over the tape but not penetrating the skin, micropore medical tape of the same size will be placed over the needles. This will remain in place until the next session.

### Assessment

The evaluation of each assessment scale will be performed by an investigator blinded to the assigned group. A baseline assessment will include Fisher grade (FG), HHS, modified Rankin Scale (mRS), NO and endothelin-1 in plasma [26]. The occurrence of delayed ischemic neurological deficit (DIND), TCD vasospasm, angiographic vasospasm and a vasospasm-related cerebral infarction will be monitored during the study period. DIND is defined as an unaccountable new focal neurological deficit lasting >2 hours with either angiography or transcranial Doppler findings [27]. TCD (Transcranial Doppler Sys, 2-channel, DWL, Germany) monitoring will be done by a skilled researcher at a certain time every other day [8]. CT angiography or conventional cerebral angiography will be performed when an abnormal increase in blood flow velocity is shown or any neurological changes occur [4,10]. If necessary, we will check whether vasospasm-related infarction has occurred using CT or magnetic resonance imaging (MRI) [8]. Modified Rankin scale will be measured after 2 weeks, and the follow-up assessment will be conducted 2 weeks after the primary endpoint in order to evaluate mortality and mRS.

### Combined treatment

All participants will receive conventional therapy in the same way. This is defined as general neurosurgical management carried out in SAH patients to stabilize the vital signs, control pain, and prevent vasospasm through fluid or medication therapy. Any use of medication will be recorded on the case report forms every day.

### Dropout criteria

Participants conforming to any of following conditions will be removed from the study: (1) more than three sessions (out of a total of 12) are not performed; (2) withdrawal of patient consent; (3) severe adverse events making the trial unsustainable; (4) worsening condition making it difficult to participate in the trial; or (5) impractical to continue with the trial, as judged by the principal investigator.

### Outcome measures

#### Primary outcome measurement

The occurrence of DIND will be compared between the study group and the control group. DIND is defined as an unaccountable new focal neurological deficit lasting ≥2 h. Any occurrence of DIND will be recorded by the physician and verified by the investigator every day.

#### Secondary outcome measurements

Secondary outcome measures are as follow:

1. Incidence of angiographic vasospasm
  Angiographic vasospasm is defined as focal or generalized reduction of cerebral arterial caliber on conventional cerebral angiogram or CT angiography confirmed by a neuroradiologist and a neurosurgeon.
2. Incidence of TCD vasospasm
  TCD vasospasm is defined as a peak systolic middle cerebral artery velocity (PCA_MCA_) of >200 cm/s and a Lindegaard ratio of >3 (Lindegaard ratio = mean velocity in the MCA/mean velocity in the ipsilateral extracranial internal carotid artery) [28-30].
3. Incidence of vasospasm-related infarct on CT or MRI
  A vasospasm-related infarct is defined as a cerebral infarction in the region of angiographic vasospasm or TCD vasospasm as shown on CT or MRI.
4. Changes in plasma NO and endothelin-1
  A blood sample (3 mL) will be taken from the brachial vein, centrifuged at 3,000 rpm for 15 min, and kept in a freezer at -70°C before analysis. The concentration of NO will be analyzed using a Total Nitric Oxide and Nitrate/Nitrate Parameter Assay Kit (R&D Systems, Inc., USA) and the level of endothelin-1 will be measured using an Endothelin-1 Quantikine ELISA Kit (R&D Systems, Inc., USA). All samples will be discarded after analysis (www.rndsystems.com).
5. Mortality at the end of treatment and 14 days after treatment (or at discharge)
6. Modified Rankin Scale (mRS) score
  Functional status is assessed with mRS score at the end of treatment and 14 days after treatment (or at discharge).

### Safety evaluation

Any adverse events or abnormalities will be recorded in the case report forms irrespective of the intervention used. Severity will be evaluated as mild, moderate, or severe, and the relation of any events to the intervention will be evaluated as not related, possibly related, or related.

If any serious adverse events occur, the intervention will be stopped immediately and appropriate action will be taken. This will be reported promptly to the institutional review board, according to the protocol.

### Sample size estimation

DIND, the primary outcome of the trial, was used to calculate the sample size. According to our pilot trial carried out in 2012, the incidence rate of DIND was 10% in the study group and 38.9% in the control group [24]. The difference in the expected incidence rate of DIND between the two groups (P_c_ - P_t_) is therefore 28.9%. To detect this difference with a two-sided 5% significance level and a power of 95%, 39 participants per intervention arm have to be randomized, allowing for a 20% dropout rate [31].

$$ n=\frac{{\left({z}_{\alpha /2}+{z}_{\beta}\right)}^2\left({p}_c{q}_c+{p}_t{q}_t\right)}{{\left({p}_c-{p}_t\right)}^2} $$

### Statistical analyses

According to the principle of intention-to-treat, all randomized participants will be analyzed using SPSS version 18.0 (SPSS Inc., Chicago, IL, USA) software. All data will be represented as mean ± standard deviation (SD) or number (%). For comparisons of two groups, an independent samples *t*-test or Mann- Whitney *U* test will be used for continuous outcomes, and a χ^2^-test or Fisher’s exact test will be used for noncontinuous outcomes. To control for possible confounding factors (for example, age, Hunt and Hess Scale, modified Rankin Scale, *etcetera*), multiple regression analysis will be performed for the comparison of two groups. Confidence limits of 95% will be calculated and *P* values of < 0.05 will be considered statistically significant.

## Discussion

In this randomized controlled trial, we aim to observe the effect of acupuncture treatment on the prevention of vasospasm after SAH. In addition to the occurrence of DIND and vasospasm, plasma levels of NO and endothelin-1 will be evaluated to identify the mechanism of action of the treatments used. Stratified block randomization will be used according to sex and severity (HHS). For the placebo control, we will use an electric stimulator without electrical flow and sham intradermal acupuncture designed not to penetrate the skin. With use of the same electrical stimulator and intradermal acupuncture method as the treatment group, better blinding of the control group is expected. Participants will be treated alone in a treatment room in order to avoid any communication with other participants.

In summary, the effect of acupuncture treatment on the prevention of vasospasm after SAH will be assessed in this trial. The placebo effect should be excluded and the trial design should allow for evaluation of the efficacy and safety of acupuncture for SAH patients.

### Trial status

Recruitment started in September 2014 and will be completed by the end of May 2016.

## Abbreviations

CT: Computed tomography
DIND: Delayed ischemic neurological deficit
FG: Fisher grade
HHS: Hunt and Hess Scale
MRI: Magnetic resonance imaging
mRS: Modified Rankin Scale
NO: Nitric oxide
SAH: Subarachnoid hemorrhage
TCD: Transcranial doppler
triple-H: Hypertensive, hypervolemic, and hemodilution
